# Phenotypic Heterogeneity in Tumor Progression, and Its Possible Role in the Onset of Cancer

**DOI:** 10.3389/fgene.2020.604528

**Published:** 2020-11-30

**Authors:** Saniya Deshmukh, Supreet Saini

**Affiliations:** Department of Chemical Engineering, Indian Institute of Technology Bombay, Mumbai, India

**Keywords:** metabolism, phenotypic heterogeneity, cancer, signaling, gene regulation

## Abstract

Heterogeneity among isogenic cells/individuals has been known for at least 150 years. Even Mendel, working on pea plants, realized that not all tall plants were identical. However, Mendel was more interested in the discontinuous variation between genetically distinct individuals. The concept of environment dictating distinct phenotypes among isogenic individuals has since been shown to impact the evolution of populations in numerous examples at different scales of life. In this review, we discuss how phenotypic heterogeneity and its evolutionary implications exist at all levels of life, from viruses to mammals. In particular, we discuss how a particular disease condition (cancer) is impacted by heterogeneity among isogenic cells, and propose a potential role that phenotypic heterogeneity might play toward the onset of the disease.

## Introduction: Phenotypic Heterogeneity in Isogenic Cells

Heterogeneity in a population has been a topic of long-standing interest in populations. Darwin was interested in small variations in a particular trait in a population. He was convinced that it was these small continuous changes in a population, which were responsible for evolutionary change (Darwin, 1859). We now understand that continuous variation can be exhibited because of a trait being a polygenic character, or because of the phenotypic variation among isogenic individuals (Dudley and Lambert, 2004). However, this was not the case in the late 19th century. On the one hand, Mendelians (led by Bateson) were convinced that small, continuous variation of a trait was irrelevant as far as evolutionary change was concerned (Bateson, 1894). It was a discontinuous variant of a trait in a population, which lead to an evolution in populations. On the other hand, Mendelians, lead by Pearson and Weldon, insisted that continuous variation was sufficient to bring about evolutionary change. The debate led to the famous, and often bitter debate between the Biometricians and Mendelians.

Although early efforts at reconciliation began in the early 20th century, it was not until Fisher’s publication in 1918, which reconciled the two sides (Fisher, 1918). The strategy adopted by Fisher was partitioning the variance in a phenotype among material causes.

**FIGURE 1:**
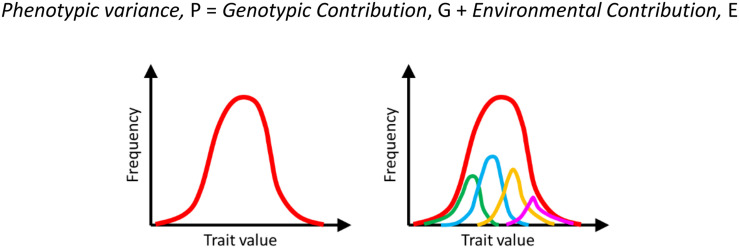
Heterogeneity in a population is a result of both genetic and environmental contributions. The overall manifestation of this heterogeneity in a population is a distribution of the phenotype on the left (red curve). This heterogeneity comprises heterogeneity due to different genotypes (indicated by green, blue, orange, and pink on the right) and the environmental noise (variation within each genotype). For example, the phenotypic distribution represented by the blue curve represents the trait distribution among isogenic individuals. This variation is because of environmental reasons.

It was recognized since Lamarck’s time that the value of a trait is dictated by not just the genetic composition of the individual, but also the environmental conditions surrounding it. Hence, an isogenic population exhibited heterogeneity (Figure 1). Here, everything that is not genetic (i.e., DNA sequence of the individual) comprises the environment of an individual.

A century later, we understand the non-genetic variation observed by Fisher much better. We now can understand the molecular causes and can appreciate the implications of intercellular variation in dictating cellular/organismic fate. The heterogeneity between the phenotype of isogenic cells plays an important role in dictating the evolutionary fate of populations much better. These non-genetic mechanisms result in phenotypic heterogeneity.

In this article, we discuss manifestations of phenotypic heterogeneity at different scales, particularly microbes and in the case of development. Last, we discuss the mechanisms of phenotypic heterogeneity which help us understand the onset and progression of a disease condition (cancer) better.

## Mechanisms of Phenotypic Heterogeneity

**FIGURE 2:**
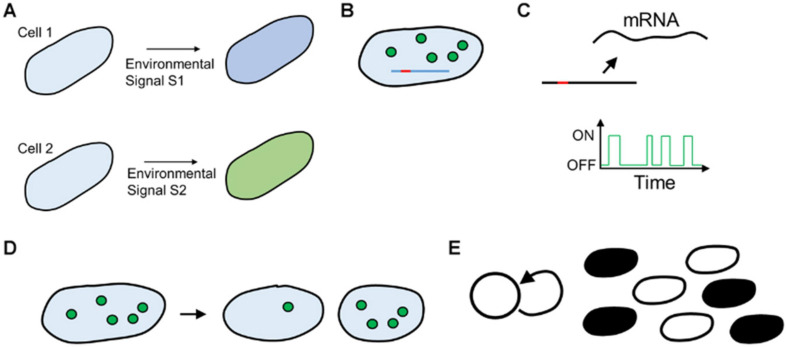
**(A)** Extrinsic cause of heterogeneity. Two isogenic cells are fed dissimilar signals because of environmental noise, leading to different responses. **(B–E)** Cell–cell heterogeneity because of intracellular noise. **(B)** Small number of Transcription Factor molecules (circles) take widely different time to search for the promoter site (red) on DNA (blue line). **(C)** Cellular processes such as transcription occur with exponentially distributed periods of bursts of activity. **(D)** Cell–cell variation because of unequal partitioning at cell division, **(E)** Feedback in cellular networks leads to all (black) or none (white) states in cellular physiology.

At a mechanistic level, why does phenotypic heterogeneity occur? Broadly, it can be classified into two categories: first, isogenic cells/individuals receiving different information from the environment can lead to different manifestations of a phenotypic trait. The second cause is cells exhibiting different phenotypes despite receiving the same environmental information. The former is called extrinsic noise, and the latter, intrinsic noise (Swain et al., 2002) (Figure 2).

Phenotypic heterogeneity in biological systems stems from several mechanistic factors.

First, the number of molecules of an average protein in a cell varies from a few dozen to several thousand (Ishihama et al., 2008; Ho et al., 2018). Thus, in the cases where the number of molecules is small in each cell, the trajectory varies from one cell to another (Rao et al., 2002).

Second, fundamental processes in cellular functioning, like transcription, occur in short bursts with large periods of relative inactivity of a promoter, giving rise of heterogeneity in a population. This manifestation means that if we take a snapshot of a population at any instant, significantly different kinetics of production of a particular protein will be observed.

Third, isogenic cells differ because of the noise due to binomial partitioning of cellular resources at the time of division (Huh and Paulsson, 2011a,b). In fact, it is argued that much of the heterogeneity attributed to gene expression stems from the noise of partitioning.

Last, cellular regulatory networks are dictated by feedback. While negative feedback homogenizes behavior, positive feedback increases the cell-cell variation in a population (Mitrophanov and Groisman, 2008; Sauro, 2009). Moreover, positive feedback in networks could lead to manifestations, where a transient commitment to one state can mean that the cell cannot come out of the state (akin to an energy minimization landscape). Such a cell is then “trapped” in that state. The complexity of regulatory networks in cells means that multiple such minima exist. This is thought to be even more important from the perspective of multicellular eukaryotic organisms, where the regulatory networks are much larger and complex as compared to bacteria and there is active communication via signaling between cells (via chemical and physical cues) of a tissue.

## Phenotypic Heterogeneity in Microbial Systems

Starting from seminal work by Delbruck, Benzer (1953), we know that at a single-cell resolution, members of an isogenic population exhibit phenotypic heterogeneity. Benzer’s work demonstrated that during infection, the λ-phage exhibits two distinct phases of life-cycle when interacting with an isogenic *E. coli* population. Delbruck quantified the distribution of burst size in a phage. See these reviews for more recent developments on this topic (Smits et al., 2006; Casadesus and Low, 2013; Ackermann, 2015).

Unlike the heterogeneity in Figure 1, where the heterogeneity is on a continuous scale, in this form of heterogeneity, two isogenic individuals exhibit two distinct binary responses. This suggests thresholding mechanisms in dictating life-cycle decisions, where a continuous distribution of a protein amount, for instance, can be converted into a phenotypic binary decision. Since Benzer’s publications, the phenomenon of phenotypic heterogeneity, in an isogenic microbial population in a well-mixed environment, has been studied in a number of contexts.

In the context of Darwinian fitness, the exhibition of heterogeneity can confer an advantage to the population. A well-studied manifestation is the persister cells in bacterial populations (Balaban et al., 2004; Dhar and McKinney, 2007; Gefen and Balaban, 2009). A small fraction of individuals in an isogenic population, caller persisters, due to their metabolic inactivity, exhibit resistance to antibiotics. Hence, should the population encounter a temporal wave of the antibiotic, these persister cells survive, and resume growth once the wave has passed. Compare this to a microbial population where every member of the population is actively growing, rendering each individual susceptible to the antibiotic. In this context, phenotypic heterogeneity aids the chances of the population surviving an environmental catastrophe. In this bet-hedging strategy, the population places individuals in different phenotypic states, and thereby, ensuring that at least one fraction of the population survives possible stress in the near future. This fitness advantage is context-dependent. In an environment where no antibiotic is encountered, persister cells will not contribute to the growth of the population. Thus, a trait of non-genetic heterogeneity, such as the commitment of a fraction of the population as persisters, is likely an adaptive response under appropriate conditions.

Such a bet-hedging strategy is used by several microbial species to counter environmental stress. Such response to anticipated stress is observed in other contexts too. In *Bacillus subtilis*, the decision to sporulate starts much before the resources run out. When exposed to starvation signals, only a fraction of cells sporulate. The remaining population switches to alternative metabolites for growth. This bet-hedging process is dictated by noise, which thus influences bacterial cell development (Veening et al., 2008). Other manifestations of this bet-hedging strategy have also been reported in other species (Galhardo et al., 2007; Sureka et al., 2008; Tiwari et al., 2010).

Recently reported manifestations of a bet-hedging strategy are more widespread than during anticipation of catastrophic events. One such manifestation has been during the transition from one carbon source to another (Ventela et al., 2003; Solopova et al., 2014). Under identical conditions, the metabolic fate of isogenic cells can have distinctly different fates which, is particularly important from the context of cancer cells (Phan et al., 2014).

In another scenario, phenotypic heterogeneity, via division of labor and cooperation among the participating phenotypic states, aids growth and survival of a microbial population (Varahan et al., 2019). A recent work (Rosenthal et al., 2018) on an isogenic population of *B. subtilis* growing in glucose demonstrated a split into two metabolic states. One converts glucose to acetate, and the other converts the accumulated acetate to acetoin (thus not enabling the accumulation of a toxic intermediate). Such division of labor is, hence, facilitated by a phenotypic heterogeneity in the population, where different parts of the population play distinct roles. The link between phenotypic heterogeneity and adaptive response has been reviewed extensively (Ackermann, 2015).

Phenotypic heterogeneity has also been observed in the context of virulence of pathogenic bacteria. In *Mycobacterium* infections, differences in the microenvironment are known to lead to divergences in the physiological states of the bacteria present in different lesions. The metabolic heterogeneity in the bacterial population, thereafter, has implications in their ability to survive stress such as drugs (Dhar et al., 2017). In *Salmonella*, phenotypic heterogeneity in the intestinal phase of infection helps the population in finding access to the niche in the body (Ellermeier and Slauch, 2007; Saini et al., 2010).

Common threads run in microbial and complex eukaryotes when studying metabolic transitions and heterogeneity. From the context of cancer, Warburg reported that cancer cells undergo aerobic glycolysis and secrete lactate (Kohler, 1973). We now know this to be almost universally true of cancer cells. In addition, the same phenomenon is also seen in yeast (De Deken, 1966) and bacteria (Wolfe, 2005; De Mey et al., 2007). The underlying principles of the logic of metabolism remain conserved across life forms, and when consuming glucose at high rates, flux channels from TCA to lactate/acetate production across bacteria, yeast, or cancer cells.

## Non-Genetic Heterogeneity in Metazoan Systems

**FIGURE 3:**
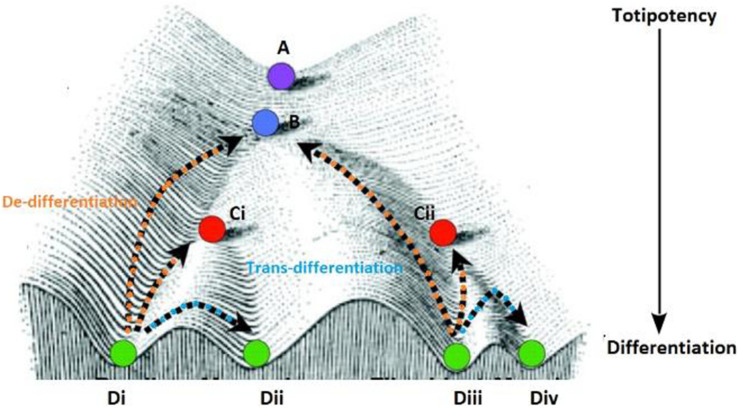
The purple circle at the top **(A)** of the Waddington landscape represents a totipotent cell with the ability to differentiate into all cell lineages **(B–D)** present in a given metazoan system. The higher positions are representative of pluripotent zones and the low-lying zones are lineage-committed zones as denoted by the arrow. As the cell divides and travels down this landscape it retains the ability to for a select number of lineages which is dependent on the path the daughter cell takes downhill (represented by the different sub-types). All the cells represented in this figure originate from the single predecessor cell starting at position **(A)** and are genetically identical however, regulation of cell fate is shaped by the position phenotypic positions specified by points **B,C (Ci–ii)** and **D (i–iv)**, a cell occupies on the landscape. Each position signifies the effect of reprogramming on genetically similar cells for the expression of a fully differentiated or committed phenotype. Some cells can get arrested in a state at higher altitudes (like position **B** or **Cii**) before the can completely differentiate and form the stem cell population, while some cells can revert to a progenitor phenotype (from the terminal phenotypic position **Di** to **Ci**) or change positions laterally (between phenotypic position **Di** to **Dii**) on this landscape.

The development of heterogeneous cell populations in multicellular eukaryotes from an embryo to a developed individual at the time of birth is a classic example of non-genetic heterogeneity. Both intrinsic and extrinsic factors dictate heterogeneity during development. The earliest representation for this diversification was proposed by Waddington, in his landscapes (Figure 3) (Waddington, 1956). The initial Waddington landscape was proposed for a developing embryo. However, our current understanding of cellular plasticity considers it to be integral to tissue regeneration in adults. In adult tissues, cells can revert to a progenitor phenotype (de-differentiation) or a mature cell can directly change phenotype (*trans*-differentiation) to recuperate after unfavorable conditions (Rajagopal and Stanger, 2016).

As discussed above, noise in biological systems can be introduced due to variation in cell-intrinsic or cell-extrinsic factors (Tsimring, 2014). Cellular noise is ubiquitous and permeates the metazoan cell hierarchy. From a single progenitor, development of all cell types takes place. The scale of this challenge varies (Number of cells in *C. elegans* ∼1000, Drosophila 10^6^, humans 10^9^). In the face of noise, how does correct cell development and differentiation take place? Wrong developmental decisions (temporally or spatially) could have fitness consequences for individuals.

This section examines the role of non-genetic heterogeneity in the normal development of two diverse multicellular systems. The first example looks at the role of heterogeneity in the holistic development of *Caenorhabditis elegans* from a single-celled zygote. While in the second example, we discuss the human reproductive system as a particular case to study variability at the level of a specialized organ system.

### Non-genetic Cellular Variability During Organismal Development

**FIGURE 4:**
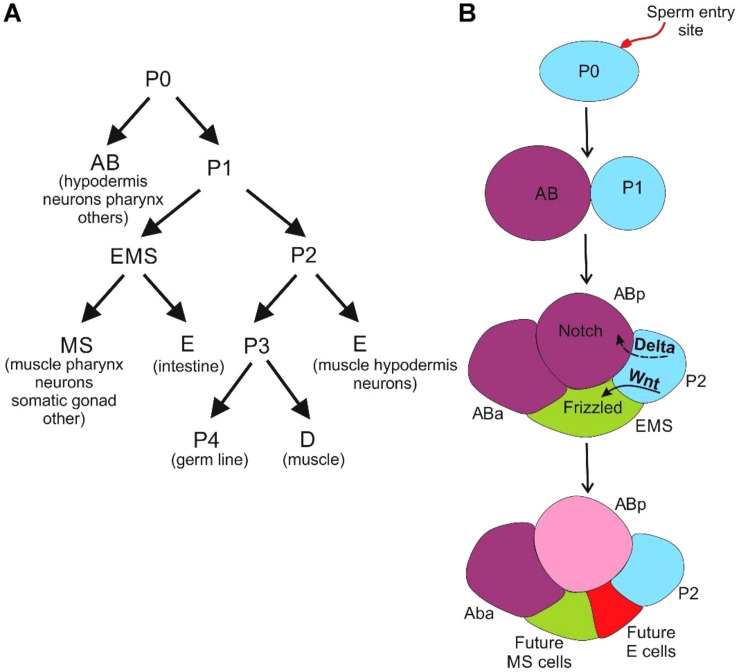
Development of *C. elegans* **(A)** the cells derived from the asymmetric cleavage of the zygote and their future lineages **(B)** the AB divides longitudinally or perpendicular to the anterior-posterior axis to produce ABa and ABp cells whereas the P1 cell divides transversely to produce founder cell EMS and a posterior stem cell (P2). The effect of positional signaling between the cells with respect to P2 cell contact, at the four-celled stage via the Notch and Wnt pathways leads to specification of EMS and ABp cells.

*Caenorhabditis elegans* is a free-living nematode with a rapid development time (3–5 days) to transition from an embryo to a fully mature adult (Frezal and Felix, 2015). An adult *C. elegans* has about 1000 somatic cells along with 1000–2000 germ cells. As the cell types in the organism are limited, a cell-by-cell reconstruction of the anatomy has been worked out to identify the lineage of each cell. *C. elegans* embryos exhibit two distinct pathways of cell fate determination. First is due to the presence of intracellular determinants in the cell (Figure 4A). And, the second pathway as the outcome of the association with the neighboring cells (Figure 4B).

We first discuss the intracellular reasons for heterogeneity. Post-fertilization, the sperm entry point polarizes the oocyte cytoplasm, and the site of localization of the male pronucleus forms the posterior end of the organism (Goldstein and Hird, 1996). The assignment of anterior and posterior poles of the embryo leads to the creation of a gradient of cellular proteins (Gotta et al., 2001). Following this, there is an asymmetrical division of cells resulting in unequally sized two cells, namely founder cell (AB) and a stem cell (P1) (Nance and Zallen, 2011). The cell polarity established by the PAR (partitioning defective) proteins mediated signaling pathway plays a vital role in the first division of the zygote occurs (Kemphues et al., 1988). The PAR proteins drive the sperm-derived centrosome to mark the posterior pole (Etemad-Moghadam et al., 1995; Guo and Kemphues, 1995; Boyd et al., 1996; Tabuse et al., 1998) while the gradient of the Gα proteins causes an imbalance in the pulling forces required for translocation of the mitotic spindle (Ajduk and Zernicka-Goetz, 2016). Another factor contributing to cellular variability is the migration of the P-granules, ribonucleoprotein complexes, which are involved in germline specification after fertilization. These granules are membrane-less organelles containing RNA (maternally expressed transcripts) and proteins associated with RNA metabolism (Marnik and Updike, 2019) and are involved in silencing the non-germline transcripts from accumulating near the nuclear pores in developing gametes. They, hence, play an essential role in the maintenance of germline identity and fertility (Rose and Gonczy, 2014; Seydoux, 2018). During the second division of cleavage, the resultant cells of unequal sizes lead to the inheritance of different cell fate determinants, enabling them to follow distinct developmental paths.

As mentioned above, the other cause of cell-cell heterogeneity is extracellular cues. The positioning of cells during the development of *C. elegans* is crucial for intercellular communication. The cells develop and differentiate according to the signals they receive from their neighbors (Figure 4B) (Evans et al., 1994; Mickey et al., 1996; Good et al., 2004; Von Stetina and Mango, 2015).

The specification of cell fates in endodermal lineage depends on interactions between P2 cell and sister cell EMS. The default state of EMS is to develop into mesoderm, which gives rise to the muscles, pharynx and other cells (Rocheleau et al., 1997; Shin et al., 1999). The association with P2 cell causes the EMS cell to polarize and rotate the spindle assembly aligning it with the anteroposterior axis. The end of the EMS cell in contact with P2 cell causes Wnt signaling asymmetry (Thorpe et al., 1997, 2000). If the gradient of Wnt signaling is equalized along with the EMS cell, the resultant daughter cells skip the endodermal fate and develop mesodermal lineages (Herman et al., 1995).

Interestingly, physical contact is not necessary for establishing Wnt asymmetry. A signaling pulse can relay this asymmetry across cell diameters which results in a small shift in the plane of cell division along the AP axis (Bischoff and Schnabel, 2006). This system of cell division and signaling induces cellular variability that aids in the assignment of distinct developmental fates (Maduro, 2010).

During the early development of *C. elegans*, unequal segregation of cellular proteins or intercellular communication gives rise to cellular variation. The process of development relies on the heterogeneity for differentiation of the multipotent predecessors to a stable cellular phenotype.

### Non-genetic Cellular Variability Within a Specialized Organ System

As discussed in the case of *C. elegans*, the communication with the extracellular environment provides essential cues to cells for development. As a metazoan embryo develops, there is organogenesis, and specific organ systems are formed. The development of organ systems requires intricate coordination of intercellular signaling within and between tissues. In this section, we consider the development of the reproductive system and subsequent gametogenesis as a model to study non-genetic heterogeneity within an organ system.

The vertebrate gonad has a unique bipotential primordium, and the nature of hormonal signals received dictate the formation of testis or ovaries, governing the phenotypic sex of the organism. The genetic sex is determined in humans by the presence or absence of the Y chromosome (Brennan et al., 2013). The male pathway is dependent on the initiation of male hormones due to gonadal expression of the Y-linked gene, sry. Subsequently, in the absence of these specific testicular hormones, the female pathway is established.

Sex determination in humans is a relatively simple process as compared to sex development. The chromosomes primarily characterize the former while the latter is a multi-parametric process involving genetic, regulatory or hormonal aspects of gonadal development. The outcomes of any abnormality in the development of external or internal genital structures are clinically classified as disorders of sex development (DSD) (Makiyan, 2016). There are multiple non-genetic factors involved in DSD, unlike in case of a chromosomal abnormality. A change in the external environment like exposure to androgens or maternal tumors can act on the bipotential gonad and cause the reversal of phenotypic sex or mosaicism leading to ambiguous development of genitalia where the hormonal factors induce variability in the phenotype of cells with similar chromosomal sex (Witchel, 2018). Ovotesticular disorder, one of the rare cases of DSD, can occur in sry-negative XX males (Ozdemir et al., 2019). The bipotential gonad develops into both, genetically identical ovarian follicles and seminiferous tubules. Potential mechanisms that could be responsible for this heterogeneity in the XX (sry-) individual could be due to the activation of testis specifying genes in the absence of sry and/or inadequate expression of pro-ovary/anti-testis genes (Witchel, 2018).

The above-cited example of a non-genetic variation in the gonadal development is evident only in case of an anomaly. However, there are sources of variation in well-developed gonads. During the process of male and female gametogenesis, cellular heterogeneity is introduced, which is discussed below.

**FIGURE 5:**
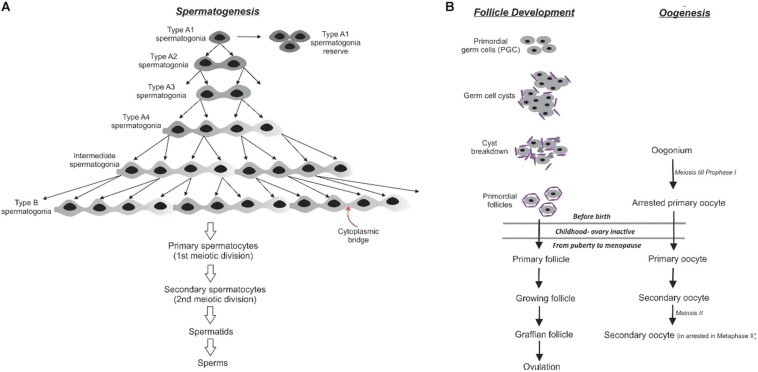
Gametogenesis in the human reproductive system: **(A)** In male spermatogenesis there is the presence of cytoplasmic bridges that connect the pre-meiotic sperm precursor cells allows the sharing of resources via cytoplasmic gradients between diploid cells from the same progenitor germ cells **(B)** in the female follicle development, germ cell cysts are connected oogonia that assist in the development of a primordial follicle.

Spermatogenesis is the production of sperm from the primordial germ cells (PGC). The PGCs get incorporated into the sex cords of male embryo and remain dormant. At puberty, the testicular Leydig cells start androgens production under the influence of the Follicle-Stimulating Hormone (FSH) and the Luteinizing Hormone (LH), which are regulated by the hypothalamus (Oduwole et al., 2018). During development, the PGCs divide to form type A1 spermatogonia which establish a pool of self-renewing stem cells (Figure 5A). Each A1 spermatogonium divides to produce an A1 spermatogonium and the type A2 spermatogonium. The A2 spermatogonia divide and progress through A3 and A4 spermatogonia stages. This final spermatogonium stage can self-renew, die, or differentiate. The differentiation into the intermediate spermatogonium confirms commitment to becoming spermatozoa, and a subsequent mitotic division forms the type B spermatogonia. They divide to generate the primary spermatocytes which enter meiosis (Dym, 1994).

Spermatogenesis occurs in the lumen of the seminiferous tubules, where the Sertoli and germ cells produce estradiol-17ß (Carreau and Hess, 2010). The spermatocytes give rise to haploid spermatids. All these different stages of developing sperms are in the physical vicinity of the Sertoli cells to draw nutrition. The peculiarity of cellular divisions in spermatogenesis is their incompleteness. As a result, a large number of cells connected by cytoplasmic bridges are formed, which allows the exchange of cytoplasmic constituents from both parental cells thus maintaining clusters of related cells with a varied heterogeneous cytoplasmic composition that are diffusing into each other forming molecular gradients. All cells do not produce all macromolecules in the same concentration as they can procure them from a neighboring connected cell.

The process of oogenesis in females leads to the formation of the ovum (Figure 5B). The spermatogenesis in human males occurs from a population of self-renewing stem cells whereas, the oogonia in the ovary are limited as they are devoid of a pool of germline stem cells. At week 6 of gestation, the PGCs arrive and colonize the developing ovary (De Felici, 2013). Upon arrival at the ovary, the PGCs enter synchronous mitotic divisions with incomplete cytokinesis, producing an excess of interconnected oogonia, which forms clusters of related cells, germline cysts (Grive and Freiman, 2015). The functions of these aggregates are not as well characterized in the mammalian systems, and evidence suggests that mitochondria could be exchanged between members of a cyst (Motta et al., 1997; Pepling, 2012). Apoptosis regulates the number of fetal germ cells forming primordial follicles in many organisms (Matova and Cooley, 2001). In human gestation, at around 16 weeks, these cysts breakdown to smaller groups of cells and most of the oocytes undergo apoptosis (Bergeron et al., 1998; Morita et al., 2001; Pepling, 2012). It is suggested that clonally obtained cysts with genetic similarities act in unison to improve oocyte quality. There is a disparity in the future of these cells, only one cell matures to form a mature oocyte while the others act as nurse cells to nourish and act as sinks for damaged cellular components and reservoirs of mitochondria for the dominant oocyte. At the time of meiotic entry, the number of female cysts could act as a determinant of the number of primordial follicles at birth (Lei and Spradling, 2013). This reserve of follicles comprises the ovarian reserve of an adult female, which is cyclically stimulated (Grive and Freiman, 2015).

The phenotypic heterogeneity is an important facet of normal metazoan development. The creation of cellular heterogeneity is a stochastic albeit important event. It creates noise which allows expansion of cellular fate evolution. In the case of early development cited in the *C. elegans*, an unequal division of cellular determinants between daughter cells and their neighboring associations are crucial in the assignment of their fate. The human reproductive system is a specialized system that develops from a group of progenitor cells, this system exploits the cellular disparity to distribute biosynthesis load amongst genetically identical cells. Thus a specialized tissue system ustilises noise for resource allocation and energy efficiency of a developing system.

Living systems are shaped by an intricate balance of deterministic laws and randomness (Monod, 1974). Control of noise is critical – as unregulated noise could cause defects too. What happens when the noise goes wrong/out of control? (Raj et al., 2010) The elimination of noise suppression leads to developmental defects and disease.

## Onset of Cancer

Tumorigenesis and tumor progression has been thought to exemplify a form of somatic representation of Darwinian evolution. Cancer cells are a clonal population with accelerated growth and exhibit intrinsic micro-heterogeneity attributed to non-genetic factors. The switch from normal to the cancerous state can be fulfilled by any means that is capable of randomly generating heterogeneity, conferring spontaneous individuality to daughter cells (Brock et al., 2009).

To be arrested in a state of cancerous growth, a cell has to “achieve” two goals. One, enter proliferative growth, and second, escape apoptosis, which was presumed to be acquired only through mutations. The resultant changes in the functioning of oncogenes or tumor-suppressing genes via mutations potentially upset the regulatory balance between proliferation and apoptosis, allowing cells to enter the state of cancerous growth.

Assumed to be driven by somatic mutations, which push the cell into a proliferative growth state, and suppress apoptosis. Avoiding proliferative growth is a particular challenge for vertebrates with their long lives, and therefore are likely to acquire a proliferative mutation. Given the mutation rates (for humans, 60 per genome per generation) (Kong et al., 2012; Segurel et al., 2014) and the number of cells in large vertebrates [humans, O(10^12^)] – it is almost a surprise that cancer is not more prevalent. Their long lives also impose a regulatory challenge where these organisms have to permit cells to proliferate when needed (e.g., an injury) and stop growing to avoid a cancerous fate. This dilemma suggests that robust mechanisms must exist in the organisms regulatory network to permit and stop proliferation, depending on intracellular and extracellular cues.

Critical among them must be a large number of inhibitory factors, which permit growth only in the strictest of conditions, presence of mitogens being one. Thus, cancer manifestation only takes place when (a) cell(s) enter proliferative growth in an uncontrolled manner, (b) the individual is unable to shed/differentiate this cells undergoing division, (c) regulatory mechanisms intended to control cell growth fail.

One of the most common manifestations of cellular proliferation is the commitment to aerobic glycolysis. Otto Warburg observed that despite the presence of oxygen cancer cells had higher glucose utilization accompanied by lactate accumulation. Warburg (1956) explained the phenomenon through dysfunctional mitochondria, perhaps through mutations. Although this was found not to be the case, research studying the onset of cancer has largely focused on somatic mutations. The idea was first proposed by Boveri, who suggested a role for chromosomal instability in the onset of cancer (Holland and Cleveland, 2009).

Irrespective of the origins, the precise reason for cellular commitment to aerobic glycolysis during cancer is not known. Aerobic glycolysis, although less efficient than mitochondrial oxidation in terms of ATP/glucose yield, permits up to 100 times faster processing of glucose (Shestov et al., 2014). It is particularly essential since tumor environments are crowded (1 cm^3^ has 10^9^ cells), and hence any opportunity to capture resources must be utilized. Warburg Effect has also been proposed to provide the necessary carbon flux for anabolic demands of rapidly proliferating cells (DeBerardinis et al., 2008; Levine and Puzio-Kuter, 2010; Cairns et al., 2011), or regeneration of NAD from NADPH (Vander Heiden et al., 2009; Lunt and Vander Heiden, 2011). Warburg effect also proposes the alteration of cellular signaling (Wellen and Thompson, 2010, 2012; Locasale and Cantley, 2011). In a different spirit of reasoning, the Warburg effect has been proposed to aid in invasiveness, by altering the tumor-stroma interface via the release of H^+^ ions (Estrella et al., 2013).

So, what are the mutations which permit cells to enter the proliferative growth state? The first class of such mutations is one which makes cell division independent of the presence of mitogens (Olmez et al., 2015; Matson and Cook, 2017). Another class of mutations is one where the late G1 cell-cycle checkpoint fails. Escaping apoptosis – this is done in normal tissues too, and the precise signal which helps the cell escape apoptosis is unique in each microenvironment. For example, in epithelia, if cells lose physical contact with neighbors, apoptosis is triggered. Thus, mutations which help the cell escape these apoptotic signals (IGF-1, Atk etc.) (Chen et al., 2017; Wang et al., 2018).

Consistent with these ideas, many theories regarding the origin of cancer involve mutations, which upset the regulatory balance in a cell. In this context, we discuss these ideas before moving to possible mechanisms where non-genetic heterogeneity leads to onset of cancer.

### Somatic Mutation Theory

In this context, several theoretical ideas regarding the acquisition of mutations and the onset of cancer have been proposed. The Two-Hit Model in 1971, through a statistical analysis of retinoblastoma of the eye, proposed that two mutations cause this cancer. The two mutations can both occur in somatic cells, or one inherited, and other in somatic cells (Knudson Jr., 1971). Boveri in his 1976 book titled “The Origin of Malignant Tumors” proposed that “the problem of tumors is a cell problem” and that cancer was due to “a certain permanent change in the chromatin complex” which, “without necessitating an external stimulus, forces the cell, as soon as it is mature, to divide again.” (Manchester, 1995). Ever since, cancer has become increasingly considered as a problem of cell proliferation due to permanent changes in the “chromatin,” a term that in Boveri’s time was already known to contain the heritable material.

These theories developed and established the view that while there might be genetic heterogeneity in a tissue, a tumor has clonal origins (Nowell, 1976). This view has been successful in the identification of precise mutations associated with several cancer types (Sawyers, 2004).

### Cancer Stem Cell Theory

Through work with stem cells and leukemia in a mice model, the cancer stem cell theory was proposed in 1994. According to this idea, cancer arises from a mutation in a stem cell (a hematopoietic stem cell in this case), which gives rise to a cancer stem cell. This cancer stem cell retains the ability for self-renewal, and also to proliferate (Lapidot et al., 1994; Rosen and Jordan, 2009).

### Epigenetic Theory

The establishment of the role of epigenetics in development and gene regulation lead to the proposal of epigenetic reasons for the onset of cancer (Feinberg et al., 2006). Hyper- and Hypomethylation of DNA were demonstrated with silencing the expression of tumor-suppressing genes and activating expression of oncogenes, respectively (Cho et al., 2000; Jones and Baylin, 2002; Sato et al., 2003).

### Tissue Organization Field Theory (TOFT)

Cancer is a tissue-based disease, and that proliferation is the default state of all cells (Soto and Sonnenschein, 2011). The tissue organization field theory (TOFT) states that carcinogenesis takes place at the tissue level of biological organization, as does normal morphogenesis. In this view, a cell is not necessarily a basic unit – for example, without interaction with the ureteric bud, kidney development will fail. Thus, tissue, not a cell, should be viewed as a basic unit of multicellular life. The second premise of TOFT is that the default state of all cells is proliferation.

For instance, when mice are “initiated” by feeding small quantities of a carcinogen, a coal tar derivative, the mice develop tumors long after this exposure (Friedewald and Rous, 1944). This is presumably caused by some change brought into the cells because of the exposure to the carcinogen. However, what was this change which increased the cellular propensity to go into a tumor state? Similar observations exist with experiments with *in vitro* cells (Kennedy et al., 1984). When irradiated with X-rays, it takes these cells many generations to become cancerous. What causes this long duration between exposure and the cells becoming cancerous? The kinetics of this process and the underlying link with the mechanisms that trigger cancer are unclear. This conundrum is laid out in Brash and Cairns (2009a, b) as:

“The prime mystery in carcinogenesis remains the very first step because it is hard to imagine how the numerous genetic changes found in cancer cells could have been produced in any cell as the result of a single exposure to a DNA-damaging agent, or why months or years should have to elapse before the effect of these changes is observed” and “…the picture that emerges from the classical studies of the epidemiology of human cancers and of experimental carcinogenesis in animals is hard to reconcile with what has been learnt about mutagenesis in simple systems such as the bacteria. Initiation seems to be far too efficient to be simply mutagenesis of certain oncogenes and suppressor genes, and the subsequent time-dependent steps are even more obscure.”

Hence, an alternate paradigm regarding the onset of cancer is needed.

## Could Cancer Onset Be Triggered by Non-Genetic Heterogeneity?

In 1932, the American geneticist Sewall Wright proposed the concept of a fitness landscape (Wright, 1932). Several representations of a landscape exist (Kaplan, 2008), in one, the genetic identity of an individual could be mapped on an *N*-dimensional space (called sequence space), where each dimension corresponds to a particular locus on the genome. The *N* + 1*th* dimension represents the fitness of the individual in a particular environment. Wright proposed that such a structure be called a fitness landscape and that among the topological features of this structure is multiple local optima of fitness.

The analogy can be extended to networks too. The *N* + 1th dimension represents the stability of the network, which can represented as inverse of the potential energy of the system. In such a representation, the *N* axis represents the amounts of the *N* regulatory proteins. Regulatory networks are highly interconnected structures, and their potential and stability have been a subject of various studies. Even the simplest regulatory/signaling network where two proteins are mutually repressing has two stable and two unstable steady states. From the perspective of this discussion, this implies that the system has multiple energy minima states available to it. In such a context, the starting point and the consequent noise has a large bearing on the eventual steady state of the system. The manifestations of this idea, in higher dimensionality, could offer many more stable steady-states for the cellular regulatory logic.

Interestingly, at the time of the proposal, the idea of fitness landscapes consisting of multiple peaks and valleys was fiercely contested by the Fisher(1941; Provine, 1986). He proposed that increased dimensionality of the landscapes decreased the probability that a particular genotype corresponded to one of the maxima or minima in all the dimensions of the landscape. Thus, while the concept of valleys and troughs was acceptable in lower dimensions, at an organismal level, the high dimensionality of the structures meant that there was only one global maximum.

In the context of cellular networks, therefore a commitment to alternate steady state, leading to a cancer phenotype remains a distinct possibility. The most common manifestation in cancer is the commitment to cell proliferation and escape from apoptosis. In order to facilitate rapid division, cancer cells commit to aerobic glycolysis. At the same time, the cells escape apoptosis. The molecular pathways dictating cellular commitment to these fates are well understood.

Before we discuss the possibility of cellular commitment to proliferation and escape apoptosis, we discuss two cases where phenotypic heterogeneity has been demonstrated to have adaptive fitness.

Rutherford and Lindquist (1998) demonstrated that a mutant *Hsp90* in Drosophila leads to phenotypic abnormalities in the development of the fly. The observation resulted from a competition for role of *Hsp90* in developmental and a cell stress chaperone. In normal conditions, Hsp90 buffers the variation in a population, which only manifests neutrally. However, when the function of Hsp90 is compromised (mutations or pharmacology), phenotypic variation manifests. Selection acts on this variation, and the selected variants continued to express the variant trait, even after the restoration Hsp90 function. This study provided evidence that genetic backgrounds, which facilitated a greater variation among individuals, were more evolvable. This phenomenon was shown to be a general manifestation of phenotypic heterogeneity across life forms (Queitsch et al., 2002).

Collins and coworkers demonstrated that phenotypic heterogeneity due to transcriptional noise could aid adaptation too (Blake et al., 2006). The authors designed an engineered promoter in yeast, and working with a variety of TATA boxes in the promoter region, demonstrated that promoter designs which exhibited greater variability in the expression of the downstream gene also conferred a greater ability to withstand acute environmental stress.

These results establish the significance of phenotypic heterogeneity and evolvability of a population. These two studies establish the concept that selection acting on a population, chooses the best available phenotype. The survival of this variant, in case of stress, provides the opportunity to a fraction of the population to pick up a mutation and “solidify” this trait.

A report from Paul Rainey’s group followed, demonstrating with *Pseudomonas*, that such phenotypic heterogeneity can be evolved “*de novo*” in a population fairly rapidly. Hence, the link between phenotypic heterogeneity and evolvability was firmly established. Other examples of noise facilitating adaptation exist (Acar et al., 2008; Cagatay et al., 2009).

If cancer can be triggered by phenotypic heterogeneity, the cellular commitment must be so that the cells escape apoptosis, differentiation, and commit to proliferation. For this to manifest, signaling pathways have to be channeled to suppress apoptosis, and metabolism has to be channeled to drive cell division. We next discuss both these facets. We start with a discussion on metabolism.

### HIF1 Mediated Feedback and Commitment to Glycolysis

It is well established across different scales of life that faster growth is supported by fermentation and not TCA, despite the lower efficiency of fermentation compared to aerobic respiration. Several ideas have been proposed to explain this, including, surface area availability (Szenk et al., 2017), protein production cost (Kafri et al., 2016), rate of release of energy. This phenomenon is known to be present in microbes (overflow metabolism), yeast (Crabtree effect), and humans (Warburg effect). Interestingly, the Warburg effect is a hallmark of cancer cells. Thus, the first step toward phenotypic heterogeneity “pushing” a cell toward cancer phenotype is a commitment toward aerobic glycolysis. For this purpose, there is active suppression of mitochondrial activity, and the glycolytic pathway is activated in order to channel greater glucose flux through them. The molecular link that facilitates this is the Hypoxia-inducible factor 1 (HIF1).

**FIGURE 6:**
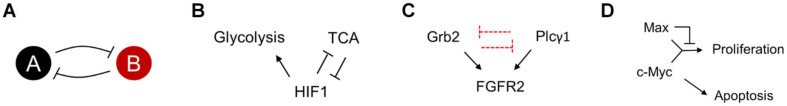
**(A)** A double negative feedback loop is inherently bistable. The steady state of the system depends on the starting position of the network in the state space. **(B–D)** Regulatory topologies of metabolism **(B)**, signaling **(C)**, and gene regulation **(D)**, which could likely have distinct steady states. The different steady states reflect cellular commitment to proliferation or lack of (or apoptosis).

HIF1 is a dimer of HIFα and HIFβ (Wang et al., 1995; Yang et al., 2005). The presence of oxygen results in the active degradation of HIFα via TCA intermediates (Chan et al., 2005). However, in low oxygen, HIF1 actively represses the expression of pyruvate dehydrogenase kinase and upregulates enzymes in glycolysis (Kim et al., 2006). This double-negative positive feedback is a hallmark of instability in the cellular regulatory network and can lead to altered commitments of individual cells among a population (Figures 6A,B).

### Grb2 and Plcγ1 Competition for FGFR2 and Cell Proliferation

Recent reports suggest that fibroblast growth factor receptor 2 (FGFR2) expressing cancer cells, which have a low abundance of the protein Gbr2, show a high abundance for metastasis. The Gbr2 and Plcγ1 (phospholipase Cγ1) in a cell compete for access to the FGFR2 protein. Reduced Gbr2 protein levels in the cell, lead to elevated access of Plcγ1, leading to downstream activation of the Atk signaling pathway, eventually leading to cell proliferation (Figure 6C) (Timsah et al., 2014, 2016). This demonstration of fluctuations in protein numbers leading to cell fate suggests that it is conceivable that healthy tissue can, via stochastic fluctuations, escape the control of growth and go into a proliferative mode of existence. The competition for an active site between two proteins constitutes the regulatory topology of a cell. In cases like the Gbr2 and Plcγ1 competition, the regulatory topology manifests as the representative of a topology in a cell.

### Dual Role of Transcription Factor Myc

Myc is one of the transcription factors which controls the expression of genes necessary for cell proliferation (Henriksson and Luscher, 1996; Roussel et al., 1996; Bouchard et al., 1998). However, the precise regulatory network dictating this activation has a more complex topology (Figure 6D). Myc, in a dimer with Max, is an activator of cell proliferation proteins. However, a dimer of Max (or a dimer of Max and one of its many partner proteins), acts as the repressor of the same genes. Thus, the precise control of proliferation or quiescence is controlled by the precise levels of these transcription factors. In contrast with its role in proliferation, Myc is also known to be a regulator of apoptosis in mammalian cells (McMahon, 2014). Myc’s role in apoptosis is achieved via the integration of a large number of cell cycle signals (Prendergast, 1999).

The key features of all the regulatory cases discussed above is the presence of bistability in the networks. One of the key characteristics of a cancer cell is proliferation. All the above networks show that control of apoptosis and proliferation is controlled via networks, which could commit to one state or the other, depending on the precise state of the system Numerous check points control cell division, and only when all fail will a cell fall into the proliferative state. Once this rare event of a cell evading cell-cycle control happens, and getting “trapped” in a proliferative state, selection acts to select a mutation which “freezes” this proliferative state.

Tumorigenesis is associated with abnormal cell proliferation, abrogation of apoptotic processes, invasiveness and metastasis. The concept of genetic determinism and non-genetic heterogeneity are intertwined in cancer physiology and progression. The genomic instability leads to genetic heterogeneity in cancer. Whether a novel genotype is the premise for a novel phenotype or vice versa remains as the inherent paradox in cancer evolution (Frank and Rosner, 2012). Thus, the variation observed within a population of clonal cells, within a tumor cannot be explained on the basis of genetic mutations alone.

## Non-Genetic Heterogeneity in Cancer

We highlighted the role of phenotypic heterogeneity in normal developmental processes. Historically, Virchow first observed pleomorphism of cancer cells within tumors establishing intratumoral heterogeneity of cellular phenotypes (Almendro et al., 2013). This finding led to a series of studies that have since demonstrated the presence of distinct subpopulations of cancer cells within tumors (Makino, 1956; Fidler, 1978; Heppner and Miller, 1983; Lawson et al., 2018; Keller and Pantel, 2019).

A novel genotype exhibits a new phenotype (Bronstein and Akil, 1990). According to the somatic mutation theory, the evolution of cancer proceeds by the acquisition of genetic changes. In recent years, there has been significant evidence claiming that new non-heritable phenotypic variants can precede genetic variants in cancer evolution (Frank and Rosner, 2012). If the phenotypic variants in a clonal population develop resistance or an advantage over other sub-populations under selective pressures, like changes in the microenvironment or drug treatment, could lead to the selection of a new genetic variant (Yang et al., 2010; Altschuler and Wu, 2010). The phenotypic heterogeneity improves the cellular response to environmental challenges during tumorigenesis and enhances the rate of evolutionary changes (Frank and Rosner, 2012). The complexity of cancer makes it difficult to state if the chronology of phenotypic and genetic variants and their exact contribution to the processes that lead to the progression of cancer.

In this section, we discuss cellular processes which contribute to the phenotypic heterogeneity among cancer cells. These facets of cellular variability are of importance in improving our understanding of cancer progression and design of therapeutic measures.

### Signal Transmission and Response

The signaling pathways create a communication web to simultaneously relay information within and between cells, connecting tissue systems to restore homeostasis within the metazoan system (Zhang and Liu, 2002; Guruharsha et al., 2012; Schrier et al., 2016; Schwartz et al., 2016). Cross-talk within the tumor microenvironment (TME) can occur through a diverse range of direct mechanisms like cell-to-cell contact via adhesion molecules, gap junctions, or indirect mechanisms through paracrine signaling by cytokines, extracellular vesicles etc. (Dominiak et al., 2020). Thus, signaling can act as a cause of and be affected due to intratumoral heterogeneity among clonal cells. The non-homogenous response to signals within the TME, can be viewed as a bet-hedging strategy. The diversity in response by malignant cells provides a chance for a fraction of the cells to evade therapy and thereafter lead to a possible relapse (Stumpf and Pybus, 2002; Kussell and Leibler, 2005). There are multiple complications in understanding cell-to-cell communication networks within the TME, as intracellular signaling within individual cells is heterogeneous. Therefore, there is no synchronization of intercellular signals and, this lag in the relay of signals introduces non-genetic heterogeneity in the TME (Thurley et al., 2018).

**FIGURE 7:**
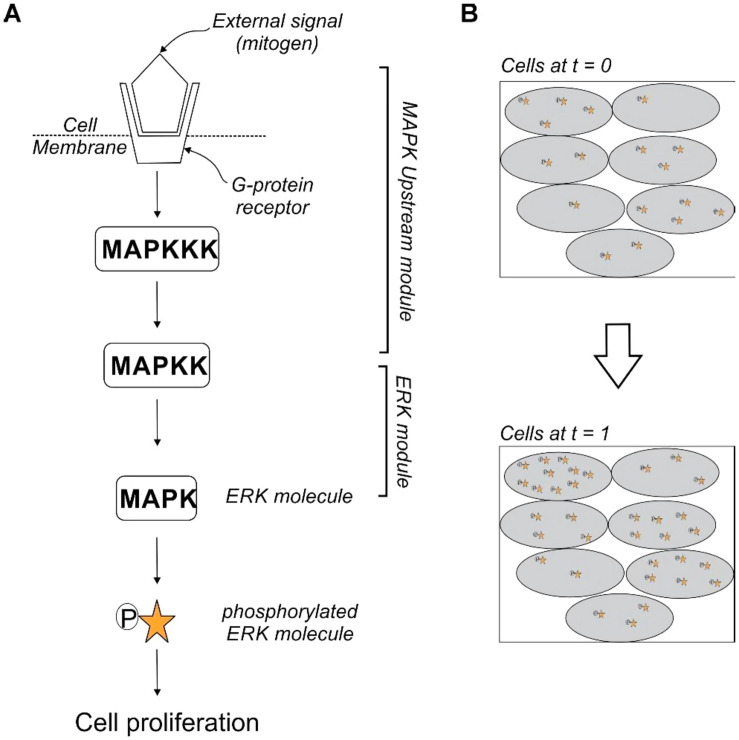
Heterogeneity of MAPK signaling **(A)** depicts the phosphorylation of the primary MAPK molecule ERK (extracellular-signal-regulated kinase) by a MAPKK (mitogen-activated protein kinase kinase). The activation of the core ERK module relays the stimulus to the nucleus for cell proliferation. Factors like ligand availability at the surface and reaction times of other reactions before the phosphorylation of ERK are the upstream module which, effect the core ERK reaction. **(B)** Cartoon of a time-lapse snapshot of cells shows that the concentration of activated ERK at *t* = 1 is dependent on the number of activated ERK molecules at *t* = 0. There is a heterogeneity in the relay of stimulus across cells in the same sub-population. This distributes the response to external stimulus within clonal cells.

The aforementioned examples illustrate phenotypic heterogeneity in different cancers and signaling pathways. The mitogen-activated protein kinase (MAPK) pathways are vital, evolutionarily conserved and link extracellular signals to fundamental processes like growth, apoptosis and differentiation (Figure 7A). Also, MAPK signaling is often the most misregulated in cancer. There are two arms of MAPK signaling, the ERK pathway and stress-activated MAPKs cascades. The ERK pathway is most well understood of the mammalian MAPK pathways and is affected in approximately one-third of all human cancers (Dhillon et al., 2007). An extracellular stimulus can activate the ERK (extracellular-signal-regulated kinase) signaling causing it to translocate to the nucleus where the signal is converted to an appropriate output and wired to the next cell. A variation in the levels of ERK activation between clonal cells in culture has been observed (Filippi et al., 2016). The different reaction rates, initial concentration of core signaling molecules and configuration of the upstream signaling cascades feeding into the ERK module could be a potential source of heterogeneity between cells (Figure 7B) (Filippi et al., 2016). These intercellular factors reduce the impact of the external signal by managing the distribution of primary MAPK activity on a cell-to-cell basis leading to signal distortion between clonal cells and generating a diverse response within the population to the same stimulus. The variability in response to external stimuli of clonal cells in a tumor distributes their risk of succumbing to immune responses of the body or therapeutic interventions.

The epidermal growth factor receptor (EGFR), from the receptor tyrosine kinases (RTKs) family, has crucial roles in Glioblastoma (GBM) development and progression (Brennan et al., 2013). RTKs pathways are crucial in the regulation of cellular signaling that controls proliferation, metabolism and response to environmental cues (Gschwind et al., 2004; Lemmon et al., 2014). The intertumoral mutational patterns of GBM are stereotypical and less heterogeneous but, striking histological variation s displayed by individual tumors (Lawrence et al., 2013; Sturm et al., 2014). Most GBM samples show the presence of different amplified RTKs, primarily, either EGFR (40–50%) or platelet-derived growth factor receptor alpha polypeptide (PDGFRA) (15%) but a small fraction show both (Furnari et al., 2015). Concurrent amplification of PDGFRA with EGFR is found to occur in 5% of GBM samples (Chakravarty et al., 2017). The RTK cell to cell variation is high. This variation redefines the tumor subpopulations based on the receptor and resultant signaling heterogeneity. The absence of uniformity in RTK introduces a high degree of redundancy in downstream interactions with Phosphoinositide3-Kinase (PI3K) and Mitogen Activated Protein Kinase (MAPK) pathways. The cell-to-cell variability due to heterogeneous RTK expression affects signaling response to RTK-inhibitors, leading to resistance to single target therapeutic approaches.

The Notch signaling pathway is involved in the determination of cellular identity and can elicit tumor suppressive or oncogenic outcomes depending on the simulation (Koch and Radtke, 2007; Ntziachristos et al., 2014). During lung development, the Notch pathway acts as a suppressor of ectopic neuroendocrine differentiation of precursor cells averting small-cell lung cancer (SCLC) (Morimoto et al., 2012; Pietanza et al., 2015). However, endogenous activation of Notch signaling causes neuroendocrine to non-neuroendocrine fate switch in 10–15% tumors. This non-neuroendocrine, Notch-active phenotype interspersed with the tumor of small-cell lung cancer is slow-growing and acts as trophic support for the neuroendocrine phenotype promoting oncogenesis (Lim et al., 2017). This phenotype is relatively chemoresistant, generating a subpopulation of persisters via activation of Notch signaling. These cells can survive chemotherapy and unless it is combined with Notch inhibition there will be inefficient tumor suppression, and relapse in the pre-clinical stages of SCLC.

There is accumulating evidence for the presence of a slow-cycling, dedifferentiated and invasive subpopulations of melanoma cells (Hugo et al., 2016; Tirosh et al., 2016; Fallahi-Sichani et al., 2017). The melanoma cells oscillate between two interchangeable phenotypes using microphthalmia-associated transcription factor (MITF)-rheostat signaling, namely, the proliferative state with high levels of MITF expression (MITFhi) or invasive phenotype with low levels of MITF (MITFlow) (Hoek et al., 2006; Zipser et al., 2011; Kemper et al., 2014). MITF maintains tumor homeostasis by regulation of cell cycle and suppression of apoptosis. The IFNγ signaling plays a crucial role in the regulation of the cytokine-mediated immune signaling. The hypoactivation of the IFNγ signaling inactivates the immunogenicity of the melanoma cells, whereas the hyperactivation creates a dedifferentiated and invasive phenotype which is a stress-induced persister population (Bai et al., 2019). This subpopulation of cells with changes in MITF levels or IFNγ signaling forms a pre-resistant cell phenotype. Together, MITF and IFNγ modulate the oscillation of cell states with constant shifts in cell phenotype of the tumor population to develop immunotherapy resistance.

### Non-homogenous Nutrient Supply and Metabolism

The cancerous cells require rapid energy and nutrition for their uncontrolled proliferation. They reform their metabolism, especially glucose, to suit their changing needs and altered microenvironment. Most cancer cells, regardless of oxygen availability, convert glucose to lactate. The glycolytic switch occurs during early carcinogenesis (Vander Heiden et al., 2009). The activation of oncogenic signaling reprograms cell metabolism, to scale up the precursors for macromolecule biosynthesis, for the accumulation of biomass during cell proliferation (Hsu and Sabatini, 2008; Schulze and Harris, 2012). Here we discuss the effect of spatial organization on metabolic reprogramming of individual cells.

The TME is an ecosystem formed by tumor and stromal cells, extracellular matrix (ECM), and secreted factors (Liotta and Kohn, 2001; Shojaei and Ferrara, 2008). The tumor tissue exhibits unique levels of cell differentiation, proliferation, vascularity, immunosuppression, and invasiveness in different pockets and contributes to the phenotypic diversity within subclonal populations (Zuazo-Gaztelu and Casanovas, 2018). The accelerated division of malignant cells causes the tumor microcapillaries to become tortuous and irregularly spaced. They develop pores of different sizes and become hyper permeable, causing the blood to leak plasma and become more viscous. These leaky vessels have reduced nutrient and oxygen-carrying capacity within different sections of the tumor (Chauhan et al., 2012; Martin et al., 2016).

**FIGURE 8:**
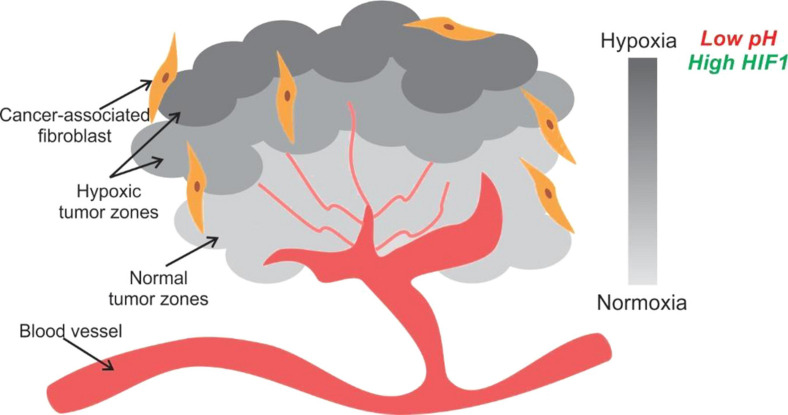
Tumor hypoxia is observed due to spatial arrangement of cells. The tumor cells away from a blood vessel are in a hypoxic and acidic environment. They generate energy for proliferation by aerobic glycolysis. Some cells in the hypoxic zone secrete HIF1 and VEGF to initiate angiogenesis for securing nutrient supply.

Unlike healthy tissue, compressed blood vessels in tumors leave large tissue volumes without blood flow and oxygen (Baish et al., 2011; Stylianopoulos and Jain, 2013). Thus, as one moves deeper into the inner mass of the tumor oxygen and nutrient supply decrease due to their distance from vascularization, making the tumor ECM heterogeneous (Figure 8) (Polyak and Weinberg, 2009; Polyak et al., 2009; Hanahan and Weinberg, 2011; Quail and Joyce, 2013).

The local irregularities and inefficiencies in the vasculature cause the initiation of unorderly angiogenesis by the tumor cells to draw nutrients. The angiogenesis is switched on by vascular endothelial growth factor (VEGF) signaling, which is upregulated by hypoxia via hypoxia-inducible factor 1α (HIF-1α) and oncogene signaling. Low extracellular pH is another outcome of poor vasculature. Hypoxia also leads to production and build-up of acidic by-products of metabolism such as lactate (Helmlinger et al., 2002; Ward and Thompson, 2012). The acidification of the microenvironment leads to higher proliferation, invasiveness and apoptosis of normal cells.

The cells in a solid tumor have the task to multiply in an environment with heterogeneous zones of hypoxia and pH. They survive by modulating their metabolism and generating diverse phenotypes to secure their resources for rapid proliferation. The level of metabolic reprogramming is fine-tuned to the local conditions like nutrient availability, oxygenation and pH. The over-production or upregulation of VEGF results in better vascularization leading to a subsequent rise in the availability of nutrients and oxygen for the tumor as a whole (Nishida et al., 2006). However, all cells do not need to go into VEGF production overdrive. The mere proximity to the over-producers of the relevant cytokines like VEGF can help non-producer cells conserve energy, and they can hitchhike at the expense of other cancer cells (Kaznatcheev et al., 2017). The free-rider phenotype has an advantage over the producer phenotype and can take over the population by harvesting more nutrients and oxygen from the neovasculature at lower energy expenditure. The different phenotypic strategies used by cells creates a metabolic heterogeneity within the tumor population.

### Mobility and Metastasis

Epithelial–mesenchymal plasticity (EMP) is a cellular mechanism, relying on the conversion between epithelium and mesenchyme in developmental milestones, like gastrulation, neural crest formation. Epithelial cells lack mobility with respect to their environment, whereas the mesenchymal cells are mobile (Larue and Bellacosa, 2005). EMP measuredly exploits this disparity in the innate properties of the two cell types during development for migration of cells and the formation of cavities. Similar physio-pathological transitions occur in cancers where there is increased motility and invasiveness during the progression of epithelial tumors.

The non-genetic phenotypic heterogeneity in cancer cells can arise due to reversible processes, epithelial-mesenchymal transition (EMT) and mesenchymal-epithelial transition (MET). The EMP is used by cancer cells for functions like metabolic reprogramming, cell proliferation, metastasis.

Cancer cells within a solid tumor exhibit widespread epithelial-mesenchymal heterogeneity and express epithelial and mesenchymal markers or co-express both and can acquire an epithelial (E), a mesenchymal (M), or one of the hybrid epithelial-mesenchymal (hybrid E/M) phenotypes respectively (Pereira et al., 2015; Hong et al., 2018; Stylianou et al., 2019). The tumor cells can exist in either of the stable phenotypes and can transition spontaneously into another state (Ruscetti et al., 2016). A series of snapshots of any clonal cell population will reveal that genetically identical cells can exist in different EMP phenotypic states over time (Tripathi et al., 2020). The dynamics of phenotypic plasticity between E and M phenotypes are affected by the initial state of the sub-population and random partitioning of parent cell biomolecules (transcription factors, regulatory proteins, miRNA and mRNAs) at cytokinesis.

Similarly, the CSC is a dynamic phenotype and can use the EMP to oscillate between the stem cell- like and differentiated phenotype (Mani et al., 2008; Zomer et al., 2013). The spatiotemporal dynamics of cells with varying EMP can lead to the formation of distinct patterns of phenotypic and functional heterogeneity of the CSCs within the tumor microenvironment (Jolly and Celia-Terrassa, 2019).

The Notch signaling pathway and EMT-inducing signals such as TGF-β together lead to distinct localization of CSCs with varying EMT phenotypes in the tumor. The Notch signaling is activated by binding of Delta or Jagged ligands on the other communicating cell. The ligand displayed by the cell decides the cellular phenotype. At low levels of both Delta and Jagged, cells exist in epithelial phenotype. The subsequent increase in the production of the ligands activates the Notch signaling, and EMT generates more number of cells in the E/M and M phenotypes.. At sites within the tumor, clusters of cells in the hybrid E/M or M phenotype are observed when Notch-Jagged signaling dominates whereas in case of cells segregate when Delta ligand is predominant (Boareto et al., 2016). Bocci et al. (2019) have modeled the diffusion of EMT-inducing signals and Notch signaling controlled non-cell autonomous switch between EMT and CSC fate decision making to reveal a distinct pattern of localization of the mesenchymal CSCs at the invasive edge, while the hybrid E/M CSCs reside in the tumor interior. The Notch-Jagged signaling stabilizes the hybrid, increases the chances of hybrid spatial proximity and expands the CSCs in a tumor (Bocci et al., 2019). The E/M hybrid is associated with higher tumor-initiating ability, a predominant trait of CSCs and drug resistance (Jia et al., 2015; Grosse-Wilde et al., 2018; Tieche et al., 2019).

### Drug Resistance

Intratumoral heterogeneity leads to the creation of different tumor subpopulations to sustain growth. A hallmark of different phenotypes of clusters of cancer subpopulations is a wide range of responses to therapeutic agents. The differential response of malignant cells can be attributed to various genetic and non-genetic sources. The CSCs were the first tumor subpopulation to be explored for resistant phenotypes (Lapidot et al., 1994). These quiescent cells help cancer acquire therapy resistance and relapse potential after the initial round of treatment (Al-Hajj et al., 2004; Fabian et al., 2013). We now view the CSCs as a tumor initiating phenotypic state which has variable markers depending on the type of cancer. The CSC hierarchies are plastic, and interconversion between the CSC and non-CSC is possible due to environmental stimuli (Batlle and Clevers, 2017).

The CSCs can be shielded from blood-borne therapies due to heterogeneous blood flow due to focal hypoxia (Martin et al., 2016). The physical sequestration of a small sub-group of tumor cells can act as seeds for relapse. There are multiple phenotypes used by persister systems to escape therapy and remain quiescent for a relapse. We highlight a few examples where the phenotypic heterogeneity of the cancer cells that helps in the acquisition of drug resistance.

Most chemotherapies target a particular receptor and its downstream effectors. There are cell signaling pathways with a heterozygous expression of surface receptors across cells. The expression of the receptors for a signaling ligand is stochastic, where some cells express either or both receptor types (Patel et al., 2014). Differential expression of ligands and cell surface receptors on a cell within a tumor builds the immunity of the tumor drug and bypasses the treatment. Mosaicism in the expression of cell receptors is widely reported, ranging from glioblastomas to non-small cell lung cancers (Hegde et al., 2013; Iqbal and Iqbal, 2014; Lee et al., 2014). Thus cells exclusively expressing a cell receptor that is not targeted by the therapeutic agent manage to tide over the treatment and cause a relapse. The signaling pathways are common between cancer and normal cells prohibiting the use of multidrug chemotherapy in many cases leaving the door open to relapse initiated by the persister pool of cells. The epithelial-mesenchymal plasticity (EMP) of cells is used by malignancies to disseminate to distant organs and in the metastasis of solid tumors. The malignant cells colonize the secondary sites and reacquire their adhesion properties. The EMP is implicated in contributing to the stemness of the tumoral mass by making it more resistant to cancer therapies (Fischer et al., 2015; Zheng et al., 2015) and evading the immune system (Kudo-Saito et al., 2009). The phenotypes created by EMP differ in their physiological properties like tumor-seeding and sensitivity to drugs (Grosse-Wilde et al., 2018; Tieche et al., 2019). The E/M hybrids form clusters of migratory cell clusters which are more resistant to apoptosis and possess an increased metastatic propensity as compared to cells with a complete mesenchymal phenotype. Thus, the EMP of cells does not only confer mobility on tumor cells but it also contributes to drug resistance.

The cancer cells undergo metabolic reprogramming to switch from OXPHOS to glycolysis which leads to increased glucose uptake to compensate for inefficient breakdown process (Bhattacharya et al., 2016; Potter et al., 2016). The activation of oncogene signaling inevitably spikes the levels of reactive oxygen species (ROS) which can cause apoptosis but are effectively managed by exploiting the inherent cell antioxidant systems activation (Irani et al., 1997; Tanaka et al., 2002). Cells use nicotinamide adenine dinucleotide phosphate (NADPH) as the antioxidant sink for ROS from the glutathione (GSH) and thioredoxin antioxidant systems. The regulation of the NADPH pool is crucial for stimulating the proliferation and survival pathways in malignant cells (Patra et al., 2013; Ciccarese and Ciminale, 2017).

There are some CSC subpopulations in tumors with higher expression of antioxidant genes and low ROS levels which show resistance to radiation therapy (Tanaka et al., 2002). For example, aldehyde dehydrogenase (ALDH) functions as an antioxidant to protect aldehydes from oxidation from byproducts generated by ROS, and the drug-tolerant persister phenotypes are ALDH high (Pribluda et al., 2015).

As illustrated by the examples above, phenotypic heterogeneity leads to the formation of a residual population post a therapeutic intervention. On account of a phenotypic variation from the other tumor cells, this subpopulation is capable of acting as seeds for relapse. There are many different routes by which some cancer cells manage to escape complete elimination. However, these persisters exploit the inherent noise in the system and use it as an asset for survival. Thus, the molecular networks of eukaryotic cells offer a myriad of opportunities for phenotypic heterogeneity to “lock” cells into phenotype, which can then lead to newer evolutionary pathways, including cancer.

## Conclusion

Historically viewed as triggered by a mutational event, recent evidence has shaped our understanding regarding non-genetic factors that can trigger cancer. In this view, cell-cell heterogeneity in gene expression leading to altered metabolic states, signaling pathways, resistance states can all “lock” a cell in a state of rapid growth. Thereafter, selection can act on this phenotype, which is then fixed by a mutational event. In this context, we present a survey of possibilities of non-genetic heterogeneity in cancer onset and progression. Experimental manifestation of these possibilities will be an important direction of future work in this area of research.

## Author Contributions

SD and SS wrote the manuscript. Both authors contributed to the article and approved the submitted version.

## Conflict of Interest

The authors declare that the research was conducted in the absence of any commercial or financial relationships that could be construed as a potential conflict of interest.
